# Computer-assisted automated synthesis. III. Synthesis of substituted N-(carboxyalkyl) amino-acid tert-butyl ester derivatives

**DOI:** 10.1155/S1463924691000330

**Published:** 1991

**Authors:** Nobuyoshi Hayashi, Tohru Sugawara, Shinji Kato

**Affiliations:** Chemistry Research Laboratories Research and Development Division Takeda Chemical Industries Ltd Juso Honmachi 2- chome, Yodogawaku Osaka 532 Japan

## Abstract

A versatile automated synthesis apparatus, equipped with a chemical artificial intelligence, was developed to prepare and isolate a wide variety of compounds. The apparatus was to the synthesis of substituted N-(carboxyalkyl)amino-acids. The apparatus [1,2] is composed of units for performing various tasks,for example reagent supply, reaction, purification and separation, each linked to a control system. All synthetic processes, including washing and drying of the apparatus after each synthetic run, were automatically performed from the mixing of the reactants to the isolation of the products as powders or crystals. The reaction of an amino-acid tertbutyl ester acetic acid salt with a 2-keto acid sodium salt produces an unstable intermediate, Schiff base, which is reduced with sodum cyanoborohydride to give a substituted N-(carboxyalkyl)aminoacid tert-butyl ester sodium salt. The equilibrium and the consecutive reactions were controlled by adding sodium cyanoborohydride using the artificial intelligence software, which contained novel kinetic equations [3] and substituent effects [4].

Substitued N-(carboxyalkyl)amino-acid tert-butyl esters, 90 derivatives, were automatically synthesized using the computerassisted automated synthesis apparatus. The syntheses were performed unattended 24 hours a day, except for supplying the raw materials, reagents and solvents. The apparatus is extremely valuable for synthesizing many derivatives of a particular compound. The configurations of the products were determined by circular dichroism measurements.


Journal of Automatic Chemistry, Vol. 13, No. 5 (September–October 1991), pp. 187–197

### Computer-assisted automated synthesis. III. Synthesis of substituted N-(carboxyalkyl) amino-acid tert-butyl ester derivatives

**Nobuyoshi Hayashi*, Tohru Sugawara and Shinji Kato**

*Chemistry Research Laboratories, Research and Development Division, Takeda Chemical Industries Ltd, Juso Honmachi 2-chome, Yodogawaku, Osaka 532, Japan*

*A versatile automated synthesis apparatus, equipped with a chemical artificial intelligence, was developed to prepare and isolate a wide variety of compounds. The apparatus was to the synthesis of substituted N-(carboxyalkyl)amino-acids. The apparatus [1,2] is composed of units for performing various tasks, for example reagent supply, reaction, purification and separation, each linked to a control system. All synthetic processes, including washing and drying of the apparatus after each synthetic run, were automatically performed from the mixing of the reactants to the isolation of the products as powders or crystals. The reaction of an amino-acid tert-butyl ester acetic acid salt with a 2-keto acid sodium salt produces an unstable intermediate, Schiff base, which is reduced with sodum cyanoborohydride to give a substituted N-(carboxyalkyl)amino-acid tert-butyl ester sodium salt. The equilibrium and the consecutive reactions were controlled by adding sodium cyanoborohydride using the artificial intelligence software, which contained novel kinetic equations [3] and substituent effects [4].*

*Substitued N-(carboxyalkyl)amino-acid tert-butyl esters, 90 derivatives, were automatically synthesized using the computer-assisted automated synthesis apparatus. The syntheses were performed unattended 24 hours a day, except for supplying the raw materials, reagents and solvents. The apparatus is extremely valuable for synthesizing many derivatives of a particular compound. The configurations of the products were determined by circular dichroism measurements.*

### Introduction

In general, organic syntheses are time-consuming; involving the preliminary estimation of the reaction conditions, mixing of the raw materials and reagents, control of the reaction conditions, concentration, extraction, purification and finally isolation of the desired products. Although some research on laboratory automation, especially for synthesis, has been attempted [5–8], an automatic apparatus for the estimation of the optimum reaction conditions, as well as control of all the synthetic processes, has remained an important objective. The authors recently reported a reaction-control methods based on the novel kinetic equations and substituent effect, which made up the computer software for the automated apparatus, and the design and construction of fully automated synthesis apparatus [1].

This paper deals with an application of the apparatus for the synthesis of substituted N-(carboxyalkyl)amino-acids—known as 'unusual amino acids' [9]. Several of these unusual amino-acids have been found to occur in nature; for example strombine, an active fish attractant extracted from *Strombus gigas* [10], and N-(carboxymethyl)-L-serine which has been isolated from asparagus shoots [11]. However, the only derivatives of N-(carboxyalkyl)amoni-acids which have been synthesized, are a series of N-(carboxymethyl)amino-acids [12].

The reaction of amino-acid tert-butyl ester acetic acid salt (1) with keto acid (2) in methanol gives an unstable intermediate. Schiff base, which is then reduced with sodium cyanoborohydride to afford the N-(carboxyalkyl)amino-acid tert-butyl ester (3). The products obtained from natural L-amino acids, all of which have an S-configuration on the chiral centre, except for cysteine and methionine, were generally a mixture of diastereoisomers [(S,R)- and (S,S)-isomers]. As the diastereoisomers were separated, their configurations were also determined by means of circular dichroism (CD) mesurement.

### Results and discussion

*Procedure for the automated synthesis*

The automated synthesis apparatus used is shown in figure 1. All synthetic processes, including washing and drying of the apparatus after a synthetic run, were automatically performed; from the mixing of reactants to

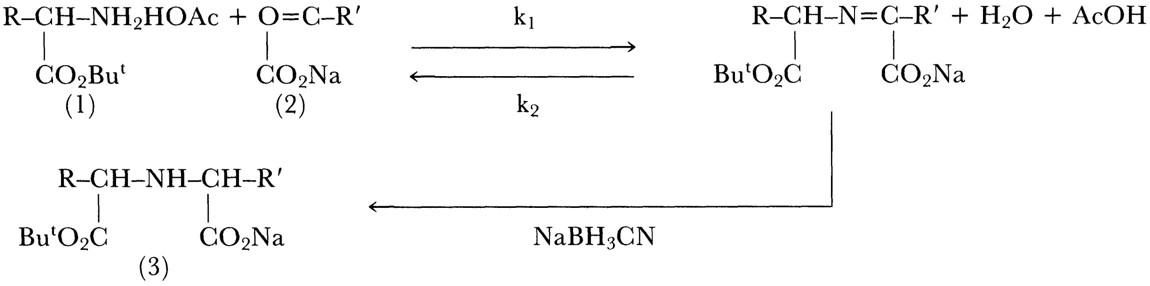

0142–0453/91 $3.00 © 1991 Taylor & Francis Ltd.

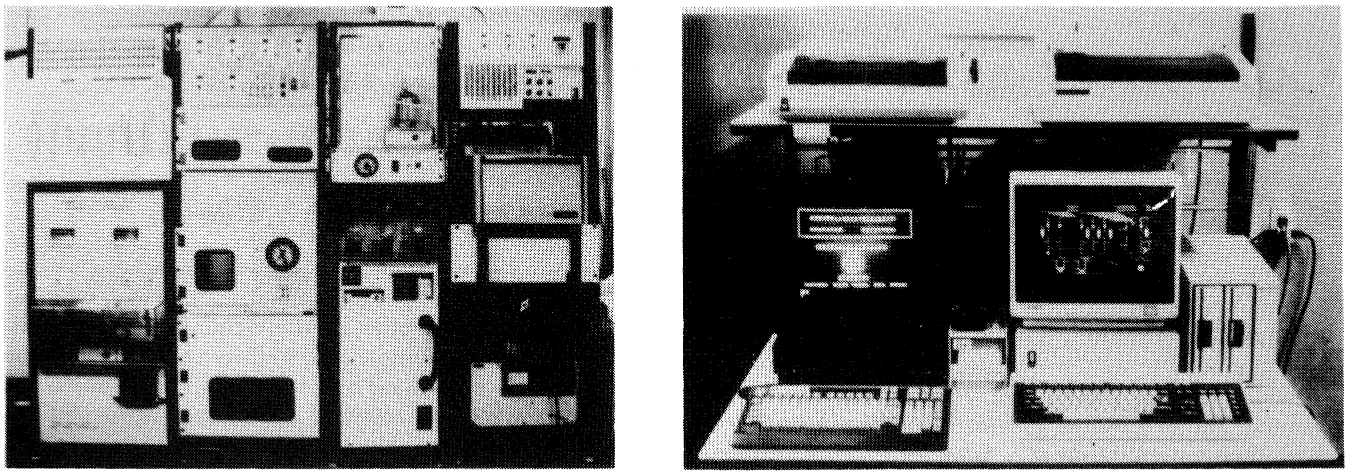

*Figure 1. The automated synthesis apparatus.*

the isolation of the products as powders. The prediction of the optimum reaction conditions and the reaction control in real time, are accomplished using the novel kinetic equations and substituent effect which make up the computer software, as previously reported [4].

As a typical automated synthesis using the apparatus, the reaction of L-leucine tert-butyl ester acetic acid salt (4) with 2-ketoisocaproic acid sodium salt (5) in methanol was accomplished in the following manner. Methanolic solutions of 4 (0·8 mol/l), 5 (0.4 mol/l), sodium cyanoborohydride (0·2 mol/l) and sodium hydroxide (0.8 mol/l; $MeOH:H_2O = 1:1$, v/v) were stored in the designated reservoirs. A flowchart showing the sequence of operations performed for a synthesis of operations performed for a synthesis is presented in figure 2. Under computer control, 5 ml of 4 and 10 ml of 5 were volumetrically measured and added to the reaction flask. The addition rate of sodium cyanoborohydride was controlled as shown in table 1.

The computer software directed the addition of sodium cyanoborohydride until the rate of decrease of the chemical yield against the reaction time reached 0·1%/min, on the basis of the kinetic equations. The reaction was then terminated 10 min after the final addition. Thus

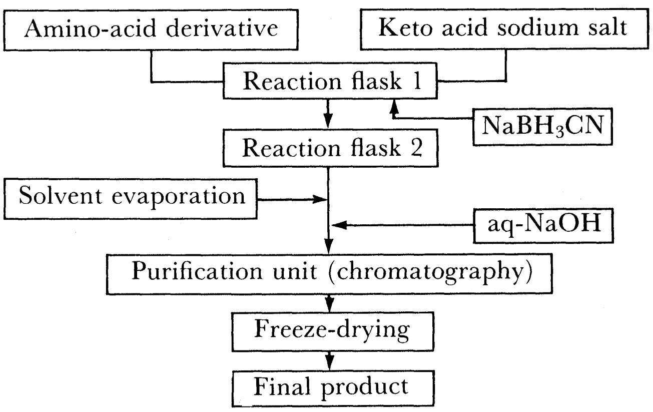

*Figure 2. The operation sequence of the automation system.*

*Table 1. The addition rate of sodium cyanoborohydride.*

| Time (min) | Addition volume (ml) |
|---|---|
| 4·7 | 4·3 |
| 13·6 | 2·9 |
| 28·5 | 2·0 |
| 52·4 | 1·4 |
| 89·3 | 0·9 |
| 145·2 | 0·6 |
| 155·2 | Finish |

the reaction was stopped after 155 min, and the resulting mixture was transferred to the concentration flask. After evaporation to dryness under reduced pressure, 10 ml of aqueous sodium hydroxide was added to the residue with stirring. The solution was then injected into the purification unit. The high performance liquid chromatographic (HPLC) chart, which was displayed on a CRT monitor during the purification stage, is presented in figure 3.

The peak at the retention time (27·2 min) was not clearly identified but it was attributed to decomposition products of the sodium cyanoborohydride and the keto acid. The two peaks having retention times of 52·3 and 66·3 min were identical to those of the diastereoisomers of N-(3-methyl-1-sodioxycarbonyl)butyl-L-leucince tert-butyl ester (6). The analytical yield calculated from the area of these two peaks was almost quantitative on the basis of

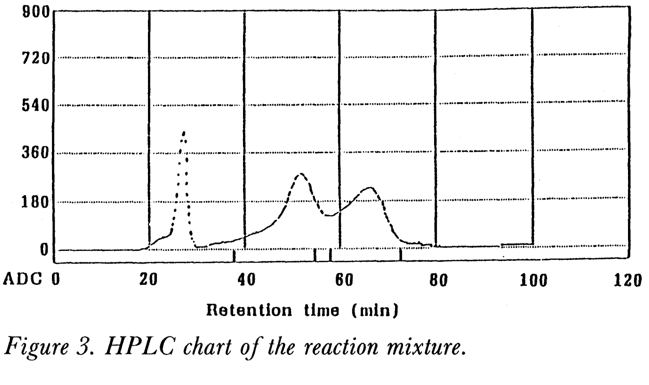

*Figure 3. HPLC chart of the reaction mixture.*

*Table 2. Chemical yields and the elemental analysis.*

| $R_1$ | $R_2$ | Yield (%) | Formula | | Calcd. (%) C | H | N | Found (%) C | H | N |
|---|---|---|---|---|---|---|---|---|---|---|
| Et | H | 64·5 | $C_{10}H_{18}NNaO_4$ | 1·5 $H_2O$ | 45·11 | 7·95 | 5·26 | 44·94 | 7·66 | 5·55 |
| | Me (M) | 65·2 | $C_{11}H_{20}NNaO_4$ | 2·0 $H_2O$ | 45·67 | 8·36 | 4·84 | 45·36 | 7·98 | 4·84 |
| | Et (A) | 76·2 | $C_{12}H_{22}NNaO_4$ | 0·4 $H_2O$ | 52·51 | 8·37 | 5·10 | 52·68 | 8·13 | 4·96 |
| | (B) | | $C_{12}H_{22}NNaO_4$ | 0·7 $H_2O$ | 51·49 | 8·43 | 5·00 | 51·64 | 8·41 | 5·05 |
| | n-Pr (A) | 73·9 | $C_{13}H_{24}NNaO_4$ | 0·3 $H_2O$ | 54·46 | 8·65 | 4·88 | 54·76 | 8·53 | 4·86 |
| | (B) | | $C_{13}H_{24}NNaO_4$ | 0·4 $H_2O$ | 54·12 | 8·66 | 4·85 | 54·17 | 8·69 | 4·78 |
| | i-Pr (A) | 59·5 | $C_{13}H_{24}NNaO_4$ | 0·4 $H_2O$ | 54·12 | 8·66 | 4·85 | 54·18 | 8·74 | 4·99 |
| | (B) | | $C_{13}H_{24}NNaO_4$ | 0·2 $H_2O$ | 54·80 | 8·63 | 4·92 | 54·93 | 8·69 | 4·98 |
| | n-Bu (A) | 78·8 | $C_{14}H_{26}NNaO_4$ | | 56·93 | 8·89 | 4·73 | 56·70 | 8·67 | 4·79 |
| | (B) | | $C_{14}H_{26}NNaO_4$ | | 56·93 | 8·89 | 4·74 | 56·83 | 9·13 | 4·79 |
| | i-Bu (A) | 78·5 | $C_{14}H_{26}NNaO_4$ | 0·2 $H_2O$ | 56·25 | 8·90 | 4·96 | 56·43 | 9·01 | 4·88 |
| | (B) | | $C_{14}H_{26}NNaO_4$ | 0·2 $H_2O$ | 56·25 | 8·90 | 4.96 | 56·26 | 8·94 | 4·63 |
| | sec-Bu (A) | 29·4 | $C_{14}H_{26}NNaO_4$ | | 56·93 | 8·87 | 4·74 | 57·19 | 9·35 | 4·40 |
| | (B) | | $C_{14}H_{26}NNaO_4$ | | 56·93 | 8·87 | 4·74 | 57·40 | 9·11 | 4·69 |
| | $(CH_2)_2CO_2Na$ (M) | 67·1 | $C_{13}H_{21}NNa_2O_6$ | | 46·85 | 6·35 | 4·20 | 45·60 | 6·65 | 4·13 |
| | $(CH_2)_3CO_2Na$ (M) | 53·4 | $C_{14}H_{23}NNa_2O_6$ | 1·5 $H_2O$ | 44·92 | 7·00 | 3·74 | 45·28 | 7·09 | 4·19 |
| n-Pr | H | 70·8 | $C_{11}H_{20}NNaO_4$ | 1·0 $H_2O$ | 48·70 | 8·17 | 5·16 | 48·20 | 7·80 | 5·31 |
| | Me (M) | 55·5 | $C_{12}H_{22}NNaO_4$ | 2·0 $H_2O$ | 47·52 | 8·64 | 4·62 | 48·00 | 8·33 | 5·17 |
| | Et (A) | 74·7 | $C_{13}H_{24}NNaO_4$ | 0·3 $H_2O$ | 54·46 | 8·65 | 4·88 | 54·77 | 8·90 | 4·90 |
| | (B) | | $C_{13}H_{24}NNaO_4$ | 0·5 $H_2O$ | 53·78 | 8·68 | 4·82 | 53·78 | 8·67 | 4·80 |
| | n-Pr (A) | 71·5 | $C_{14}H_{26}NNaO_4$ | 0·3 $H_2O$ | 55·91 | 8·91 | 4·66 | 56·04 | 9·16 | 4·60 |
| | (B) | | $C_{14}H_{26}NNaO_4$ | 0·5 $H_2O$ | 55·25 | 8·94 | 4·60 | 54·97 | 9·24 | 4·57 |
| | i-Pr (A) | 59·8 | $C_{14}H_{26}NNaO_4$ | 0·5 $H_2O$ | 55·25 | 8·94 | 4·60 | 55·40 | 8·88 | 4·70 |
| | (B) | | $C_{14}H_{26}NNaO_4$ | 0·4 $H_2O$ | 55·58 | 8·93 | 4·63 | 55·58 | 9·04 | 4·55 |
| | n-Bu (A) | 75·9 | $C_{15}H_{28}NNaO_4$ | 0·2 $H_2O$ | 57·56 | 9·15 | 4·48 | 57·82 | 9·20 | 4·48 |
| | (B) | | $C_{15}H_{28}NNaO_4$ | 0·2 $H_2O$ | 57·56 | 9·15 | 4·48 | 57·85 | 8·91 | 4·62 |
| | i-Bu (A) | 77·6 | $C_{15}H_{28}NNaO_4$ | 0·4 $H_2O$ | 56·91 | 9·17 | 4·42 | 56·94 | 9·03 | 4·50 |
| | (B) | | $C_{15}H_{28}NNaO_4$ | 0·4 $H_2O$ | 56·91 | 9·17 | 4·42 | 57·03 | 9·22 | 4·60 |
| | sec-Bu (A) | 37·0 | $C_{15}H_{28}NNaO_4$ | 0·8 $H_2O$ | 55·82 | 9·24 | 4·34 | 55·51 | 9·01 | 4·86 |
| | (B) | | $C_{15}H_{28}NNaO_4$ | 0·4 $H_2O$ | 56·91 | 9·17 | 4·43 | 56·52 | 8·99 | 4·82 |
| | $(CH_2)_2CO_2Na$ (M) | 60·0 | $C_{14}H_{23}NNa_2O_6$ | 2.0 $H_2O$ | 43·86 | 7·09 | 3·65 | 43·42 | 6·86 | 3·57 |
| | $(CH_2)_3CO_2Na$ (A) | 55·7 | $C_{15}H_{25}NNa_2O_6$ | 2·0 $H_2O$ | 45·34 | 7·36 | 3·52 | 45·48 | 7·05 | 3·57 |
| | (B) | | $C_{15}H_{25}NNa_2O_6$ | 1·5 $H_2O$ | 46·39 | 7·27 | 3·61 | 46·36 | 7·03 | 3·86 |
| i-Pr | H | 84·1 | $C_{11}H_{20}NNaO_4$ | 0·5 $H_2O$ | 50·37 | 8·07 | 5·34 | 50·08 | 7·88 | 5·59 |
| | Me (M) | 52.8 | $C_{12}H_{22}NNaO_4$ | 1·0 $H_2O$ | 50·52 | 8·48 | 4·91 | 50·02 | 8·15 | 5·01 |
| | Et (M) | 67·6 | $C_{13}H_{24}NNaO_4$ | 1·0 $H_2O$ | 52·16 | 8·75 | 4·68 | 52·26 | 8·36 | 4·75 |
| | n-Pr (M) | 68·1 | $C_{14}H_{26}NNaO_4$ | 1·0 $H_2O$ | 53·66 | 9·01 | 4·47 | 54·07 | 8·67 | 4·45 |
| | i-Pr (M) | 56·0 | $C_{14}H_{26}NNaO_4$ | 1·5 $H_2O$ | 52·16 | 9·07 | 4·34 | 51·94 | 8·75 | 4·72 |
| | n-Bu (A) | 75·6 | $C_{15}H_{28}NNaO_4$ | 0·7 $H_2O$ | 55·96 | 9·20 | 4·35 | 56·08 | 8·92 | 4·27 |
| | (B) | | $C_{15}H_{28}NNaO_4$ | 0·7 $H_2O$ | 55·96 | 9·20 | 4·35 | 55·86 | 9·30 | 4·07 |
| | i-Bu (A) | 76·5 | $C_{15}H_{28}NNaO_4$ | 1·0 $H_2O$ | 55·03 | 9·24 | 4·28 | 55·27 | 9·14 | 4·38 |
| | (B) | | $C_{15}H_{28}NNaO_4$ | 1·0 $H_2O$ | 55·03 | 9·24 | 4·28 | 55·01 | 9·34 | 4·37 |
| | sec-Bu (M) | 29·1 | $C_{15}H_{28}NNaO_4$ | 1·4 $H_2O$ | 53·84 | 9·28 | 4·19 | 54·12 | 8·99 | 4·52 |
| | $(CH_2)_2CO_2Na$ (M) | 66·1 | $C_{14}H_{23}NNa_2O_6$ | 2·0 $H_2O$ | 43·86 | 7·10 | 3·65 | 44·29 | 6·93 | 3·78 |
| | $(CH_2)_3CO_2Na$ (M) | 73·6 | $C_{15}H_{25}NNa_2O_6$ | 3·5 $H_2O$ | 42·45 | 7·60 | 3·30 | 42·10 | 7·63 | 3·32 |
| n-Bu | H | 62·5 | $C_{12}H_{22}NNaO_4$ | 1·0 $H_2O$ | 50·52 | 8·48 | 4·91 | 50·62 | 8·37 | 5·27 |
| | Me (M) | 53·5 | $C_{13}H_{24}NNaO_4$ | 1·0 $H_2O$ | 52·16 | 8·75 | 4·68 | 52·61 | 8·64 | 4·78 |
| | Et (M) | 69·6 | $C_{14}H_{26}NNaO_4$ | 0·8 $H_2O$ | 54·28 | 8·98 | 4·52 | 54·34 | 9·30 | 4·46 |
| | n-Pr (M) | 65·5 | $C_{15}H_{28}NNaO_4$ | 0·8 $H_2O$ | 55·64 | 9·21 | 4·44 | 55·76 | 9·01 | 4·29 |
| | i-Pr (M) | 59·3 | $C_{15}H_{28}NNaO_4$ | 1·1 $H_2O$ | 54·73 | 9·25 | 4·25 | 54·89 | 8·72 | 4·40 |
| | n-Bu (M) | 74·3 | $C_{16}H_{30}NNaO_4$ | 0·5 $H_2O$ | 57·81 | 9·40 | 4·21 | 57·80 | 9·19 | 4·16 |
| | i-Bu (M) | 75·5 | $C_{16}H_{30}NNaO_4$ | 0·5 $H_2O$ | 57·81 | 9·40 | 4·21 | 57·75 | 9·04 | 4·14 |
| | sec-Bu (M) | 27·3 | $C_{16}H_{30}NNaO_4$ | 1·0 $H_2O$ | 56·29 | 9·45 | 4·10 | 56·23 | 9·06 | 4·20 |
| | $(CH_2)_2CO_2Na$ (M) | 61·9 | $C_{15}H_{15}NNa_2O_6$ | 1·0 $H_2O$ | 47·49 | 7·17 | 3·69 | 47·51 | 6·91 | 3·77 |
| | $(CH_2)_3CO_2Na$ (M) | 62·9 | $C_{16}H_{27}NNa_2O_6$ | 1·5 $H_2O$ | 47·76 | 7·51 | 3·48 | 47·98 | 7·38 | 3·53 |
| i-Bu | H | 63·8 | $C_{12}H_{22}NNaO_4$ | 0·5 $H_2O$ | 52·16 | 8·39 | 5·07 | 51·73 | 8·15 | 4·91 |
| | Me (M) | 54·3 | $C_{13}H_{24}NNaO_4$ | 1·0 $H_2O$ | 52·16 | 8·75 | 4·68 | 52·15 | 8·56 | 4·52 |
| | Et (M) | 70·1 | $C_{14}H_{26}NNaO_4$ | 1·0 $H_2O$ | 53·66 | 9·01 | 4·47 | 53·45 | 8·79 | 4·58 |
| | n-Pr (M) | 68·9 | $C_{15}H_{28}NNaO_4$ | 1·0 $H_2O$ | 55·03 | 9·24 | 4·28 | 55·43 | 9·10 | 4·32 |
| | i-Pr (M) | 61·5 | $C_{15}H_{28}NNaO_4$ | 1·0 $H_2O$ | 55·03 | 9·24 | 4·28 | 55·03 | 9·20 | 4·41 |

*Table 2 (continued).*

| $R_1$ | $R_2$ | Yield (%) | Formula | Calcd. (%) C | Calcd. (%) H | Calcd. (%) N | Found (%) C | Found (%) H | Found (%) N |
|---|---|---|---|---|---|---|---|---|---|
| | n-Bu (A) | 74·9 | $C_{16}H_{30}NNaO_4$ 0·7 $H_2O$ | 57·19 | 9·41 | 4·17 | 57·24 | 9·31 | 4·34 |
| | (B) | | $C_{16}H_{30}NNaO_4$ 0·7 $H_2O$ | 57·19 | 9·41 | 4·17 | 57·32 | 9·22 | 4·36 |
| | i-Bu (A) | 72·5 | $C_{16}H_{30}NNaO_4$ 1·0 $H_2O$ | 56·29 | 9·45 | 4·10 | 56·18 | 9·46 | 4·26 |
| | (B) | | $C_{16}H_{30}NNaO_4$ 1·0 $H_2O$ | 56·29 | 9·45 | 4·10 | 56·32 | 9·58 | 4·01 |
| | sec-Bu (M) | 32·7 | $C_{16}H_{30}NNaO_4$ 1·0 $H_2O$ | 56·29 | 9·45 | 4·10 | 56·33 | 9·40 | 4·21 |
| | $(CH_2)_2CO_2Na$ (M) | 60·2 | $C_{15}H_{25}NNaO_6$ 1·0 $H_2O$ | 47·49 | 7·17 | 3·69 | 47·59 | 7·01 | 3·44 |
| | $(CH_2)_3CO_2Na$ (M) | 61·1 | $C_{16}H_{27}NNaO_6$ 1·5 $H_2O$ | 47·76 | 7·51 | 3·48 | 47·35 | 7·61 | 3·53 |
| sec-Bu | H | 68·2 | $C_{12}H_{22}NNaO_4$ 0·8 $H_2O$ | 50·87 | 8·40 | 4·95 | 50·92 | 8·16 | 4·90 |
| | Me (M) | 58·9 | $C_{13}H_{24}NNaO_4$ 1·0 $H_2O$ | 52·16 | 8·75 | 4·68 | 52·21 | 8·82 | 4·76 |
| | Et (A) | 66·8 | $C_{14}H_{26}NNaO_4$ 0·7 $H_2O$ | 54·60 | 8·97 | 4·55 | 54·90 | 9·15 | 4·67 |
| | (B) | | $C_{14}H_{26}NNaO_4$ 0·4 $H_2O$ | 55·58 | 8·93 | 4·63 | 55·68 | 9·13 | 4·69 |
| | n-Pr (A) | 75·4 | $C_{15}H_{28}NNaO_4$ 0·3 $H_2O$ | 57·23 | 9·16 | 4·45 | 57·36 | 9·28 | 4·35 |
| | (B) | | $C_{15}H_{28}NNaO_4$ 0·4 $H_2O$ | 56·91 | 9·17 | 4·42 | 57·23 | 9·02 | 4·35 |
| | i-Pr (A) | 56·6 | $C_{15}H_{28}NNaO_4$ 0·2 $H_2O$ | 57·56 | 9·15 | 4·48 | 57·64 | 8·96 | 4·52 |
| | (B) | | $C_{15}H_{28}NNaO_4$ 0·3 $H_2O$ | 57·23 | 9·16 | 4·45 | 57·29 | 9·18 | 4·64 |
| | n-Bu (A) | 78·2 | $C_{16}H_{30}NNaO_4$ 0·5 $H_2O$ | 57·81 | 9·40 | 4·21 | 58·01 | 9·30 | 4·18 |
| | (B) | | $C_{16}H_{30}NNaO_4$ | 59·42 | 9·35 | 4·33 | 59·08 | 9·07 | 4·29 |
| | i-Bu (A) | 78·8 | $C_{16}H_{30}NNaO_4$ 0·3 $H_2O$ | 58·45 | 9·38 | 4·26 | 58·68 | 9·31 | 4·49 |
| | (B) | | $C_{16}H_{30}NNaO_4$ | 59·42 | 9·35 | 4·33 | 59·14 | 9·27 | 4·51 |
| | sec-Bu (A) | 25·2 | $C_{16}H_{30}NNaO_4$ 0·4 $H_2O$ | 58·13 | 9·39 | 4·24 | 58·41 | 9·27 | 4·39 |
| | (B) | | $C_{16}H_{30}NNaO_4$ 0·4 $H_2O$ | 58·13 | 9·39 | 4·24 | 58·43 | 9·33 | 4·41 |
| | $(CH_2)_2CO_2Na$ (A) | 70·7 | $C_{15}H_{25}NNa_2O_6$ 0·5 $H_2O$ | 48·65 | 7·08 | 3·78 | 48·45 | 7·15 | 3·78 |
| | (B) | | $C_{15}H_{25}NNa_2O_6$ 0·5 $H_2O$ | 48·65 | 7·08 | 3·78 | 48·72 | 7·05 | 4·00 |
| | $(CH_2)_3CO_2Na$ (A) | 65·3 | $C_{16}H_{27}NNa_2O_6$ 2·0 $H_2O$ | 46·71 | 7·60 | 3·40 | 46·74 | 7·58 | 3·39 |
| | (B) | | $C_{16}H_{27}NNa_2O_6$ 1·5 $H_2O$ | 47·76 | 7·51 | 3·48 | 48·01 | 7·43 | 3·42 |
| $Bu^tOCH_2$ | H | 58·0 | $C_{13}H_{24}NNaO_5$ 0·5 $H_2O$ | 50·97 | 8·23 | 4·57 | 50·30 | 8·09 | 4·88 |
| | Me (M) | 53·1 | $C_{14}H_{26}NNaO_5$ 0·9 $H_2O$ | 51·33 | 8·55 | 4·28 | 51·39 | 8·78 | 4·29 |
| | Et (M) | 64·3 | $C_{15}H_{28}NNaO_5$ 0·5 $H_2O$ | 53·88 | 8·74 | 4·19 | 53·59 | 8·52 | 4·13 |
| | n-Pr (M) | 72·3 | $C_{16}H_{30}NNaO_5$ 1·0 $H_2O$ | 53·77 | 9·02 | 3·92 | 53·79 | 8·83 | 3·98 |
| | i-Pr (M) | 53·1 | $C_{16}H_{30}NNaO_5$ 1·5 $H_2O$ | 52·45 | 9·08 | 3·82 | 52·73 | 8·77 | 3·91 |
| | n-Bu (A) | 76·3 | $C_{17}H_{32}NNaO_5$ 0·8 $H_2O$ | 55·51 | 9·21 | 3·81 | 55·43 | 8·90 | 3·81 |
| | (B) | | $C_{17}H_{32}NNaO_5$ 0·8 $H_2O$ | 55·51 | 9·21 | 3·81 | 55·62 | 9·03 | 3·42 |
| | i-Bu (A) | 75·5 | $C_{17}H_{32}NNaO_5$ 1·3 $H_2O$ | 54·18 | 9·26 | 3·72 | 54·20 | 8·93 | 3·65 |
| | (B) | | $C_{17}H_{32}NNaO_5$ 1·3 $H_2O$ | 54·18 | 9·26 | 3·72 | 54·27 | 9·06 | 3·82 |
| | sec-Bu (M) | 24·8 | $C_{17}H_{32}NNaO_5$ 1·5 $H_2O$ | 53·67 | 9·27 | 3·68 | 53·23 | 8·90 | 3·51 |
| | $(CH_2)_2CO_2Na$ (M) | 53·7 | $C_{16}H_{27}NNa_2O_7$ 1·5 $H_2O$ | 45·53 | 7·22 | 3·34 | 45·61 | 7·29 | 3·87 |
| | $(CH_2)_3CO_2Na$ (M) | 54·8 | $C_{17}H_{29}NNa_2O_7$ 2·0 $H_2O$ | 46·25 | 7·53 | 3·17 | 46·20 | 8·00 | 3·49 |
| Me–CH– | H | 53·3 | $C_{14}H_{26}NNaO_5$ 1·8 $H_2O$ | 50·23 | 8·61 | 4·18 | 50·39 | 8·38 | 4·27 |
| \| | Me (M) | 58·4 | $C_{15}H_{28}NNaO_5$ 1·5 $H_2O$ | 51·12 | 8·87 | 3·97 | 51·36 | 8·89 | 4·15 |
| $OBu^t$ | Et (A) | 77·2 | $C_{16}H_{30}NNaO_5$ 0·9 $H_2O$ | 54·04 | 9·01 | 3·94 | 54·13 | 8·87 | 3·98 |
| | (B) | | $C_{16}H_{30}NNaO_5$ 1·2 $H_2O$ | 53·23 | 9·05 | 3·88 | 53·49 | 8·99 | 3·98 |
| | n-Pr (M) | 67·6 | $C_{17}H_{32}NNaO_5$ 1·5 $H_2O$ | 53·67 | 9·27 | 3·68 | 53·66 | 9·14 | 3·65 |
| | i-Pr (M) | 52·8 | $C_{17}H_{32}NNaO_5$ 1·3 $H_2O$ | 54·18 | 9·25 | 3·72 | 54·22 | 9·23 | 3·87 |
| | n-Bu (M) | 72·6 | $C_{18}H_{34}NNaO_5$ 0·8 $H_2O$ | 56·62 | 9·40 | 3·67 | 56·86 | 9·43 | 3·58 |
| | i-Bu (M) | 75·6 | $C_{18}H_{34}NNaO_5$ 1·3 $H_2O$ | 55·31 | 9·44 | 3·58 | 55·06 | 9·41 | 3·96 |
| | sec-Bu (M) | 25·1 | $C_{18}H_{34}NNaO_5$ 1·9 $H_2O$ | 53·82 | 9·48 | 3·49 | 53·84 | 9·19 | 3·71 |
| | $(CH_2)_2CO_2Na$ (M) | 55·2 | $C_{17}H_{29}NNa_2O_7$ 1·2 $H_2O$ | 47·81 | 7·41 | 3·28 | 47·87 | 7·31 | 3·41 |
| | $(CH_2)_3CO_2Na$ (M) | 57·3 | $C_{18}H_{31}NNa_2O_7$ 1·5 $H_2O$ | 48·42 | 7·67 | 3·13 | 48·63 | 8·08 | 3·04 |
| $CCH_2$– | H | 43·5 | $C_{14}H_{24}NNaO_6$ 0·5 $H_2O$ | 49·76 | 7·58 | 4·14 | 49·77 | 7·50 | 4·22 |
| \| | Me (M) | 46·2 | $C_{15}H_{26}NNaO_6$ 1·0 $H_2O$ | 50·41 | 7·90 | 3·92 | 50·27 | 8·03 | 4·05 |
| $O_2Bu^t$ | Et (M) | 60·4 | $C_{16}H_{28}NNaO_6$ 0·9 $H_2O$ | 51·99 | 8·13 | 3·79 | 52·02 | 8·10 | 3·98 |
| | n-Pr (M) | 58·2 | $C_{17}H_{30}NNaO_6$ 1·0 $H_2O$ | 52·98 | 8·37 | 3·63 | 52·89 | 8·02 | 3·54 |
| | i-Pr (M) | 52·3 | $C_{17}H_{30}NNaO_6$ 1·5 $H_2O$ | 51·77 | 8·43 | 3·55 | 52·02 | 8·19 | 3·91 |
| | n-Bu (M) | 73·4 | $C_{18}H_{32}NNaO_6$ 0·9 $H_2O$ | 54·37 | 8·57 | 3·52 | 54·56 | 8·32 | 3·58 |
| | i-Bu (M) | 72·2 | $C_{18}H_{32}NNaO_6$ 1·5 $H_2O$ | 52·93 | 8·64 | 3·43 | 52·97 | 8·44 | 3·52 |
| | sec-Bu (M) | 25·4 | $C_{18}H_{32}NNaO_6$ 1·4 $H_2O$ | 53·16 | 8·63 | 3·44 | 53·27 | 8·18 | 3·79 |
| | $(CH_2)_2CO_2Na$ (M) | 53·1 | $C_{17}H_{27}NNa_2O_8$ 1·5 $H_2O$ | 45·74 | 6·77 | 3·13 | 45·63 | 6·55 | 3·43 |
| | $(CH_2)_3CO_3Na$ (M) | 49·3 | $C_{18}H_{29}NNa_2O_8$ 2·5 $H_2O$ | 45·09 | 7·35 | 2·92 | 45·40 | 7·40 | 2·92 |

$R_1$: $R_1$–CH($NH_2$)COOH, $R_2$: $R_2$–COCOOH, A and B: separated diastereoisomers, Yield: the combined yield of diastereoisomers, M: a mixture of A and B.

*Table 3. NMR spectra of substituted N-(carboxyalkyl)aminobutyric acid tert-butyl esters.*

| R | Bu$^t$ | -CH- | -NHCHR | Other protons (Et and R) |
|---|---|---|---|---|
| H | 1·49 (s) | 3·19, 3·22 (dd, J = 5·86, 6·85) 2H, | 3·10, 3·18 dd, J = 16·5, 18·6) | 0·91 (3H, t, J = 7·51), 1·62–1·79 (2H, m) |
| Me (M) | 1·48 (s) 1·49 (s) | 3·06–3·27 (2H, m) | | 0·90, 0·92 (3H, each t, J = 7·51), 1·216, 1·224 (3H, each d, J = 6·93), 1·58–1·78(2H, c) |
| Et (A) | 1·49 (s) | 3·09 (t, J = 6·69) | 2·94, 2·97 (dd, J = 5·77, 7·42) | 0·89 (3H, t, J = 7·42), 0·92 (3H, t, J = 7·59) 1·50–1·78 (4H, m) |
| (B) | 1·48 (s) | 3·17, 3·19 (dd, J = 5·12, 7·26) | 2·99, 3·02 (dd, J = 6·03, 6·85) | 0·89 (3H, t, J = 7·42), 0·90 (3H, t, J = 7·42), 1·52–1·78 (4H, m) |
| n-Pr (A) | 1·49 (s) | 3·05 (t, J = 6·93) | 3·01 (t, J = 6·60) | 0·91 (3H, t, J = 7·10), 0·92 (3H, t, J = 6·93), 1·24–1·42 (2H, m), 1·59–1·76 (2H, m), 1·43–1·59 (2H, m) |
| (B) | 1·48 (s) | 3·17, 3·19 (dd, J = 5·11, 7·26) | 3·06 (t, J = 6·43) | 0·90 (3H, t, J = 7·43), 0·91 (3H, t, J = 7·26), 1·62–1·81 (2H, m), 1·24–1·42 (2H, m), 1·47–1·62 (2H, m) |
| i-Pr (A) | 1·50 (s) | 3·02 (t, J = 7·01) | 2·76 (d, J = 5·94) | 0·92, 0·95 (6H, each d, J = 6·92), 0·93 (3H, t, J = 7·51), 1·59–1·75 (2H, m), 1·75–1·90 (1H, m) |
| (B) | 1·48 (s) | 3·13, 3·15 (dd, J = 4·95, 9·42) | 2·83 (d, J = 5·61) | 0·90 (3H, t, J = 7·67), 0·91, 0·93 (6H, each d, J = 6·92), 1·58–1·79 (2H, m), 1·79–1·94 (1H, m) |
| n-Bu (A) | 1·50 (s) | 3·09 (t, J = 6·77) | 3·03 (t, J = 6·60) | 0·88 (3H, t, J = 7·10), 0·92 (3H, t, J = 7·59), 1·23–1·38 (4H, m), 1·50–1·75 (4H, m) |
| (B) | 1·48 (s) | 3·18, 3·21 (dd, J = 5·12, 7·26) | 3·05 (t, J = 6·43) | 0·89 (3H, t, J = 6·85), 0·90 (3H, t, J = 7·51), 1·20–1·35 (4H, m), 1·55–1·80 (4H, m) |
| i-Bu (A) | 1·50 (s) | 3·06 3·08 (dd, J = 5·12, 6·77) | 3·04 (t, J = 6·19) | 0·91, 0·92 (6H, each d, J = 6·43), 0·92 (3H, t, J = 7·26), 1·40 (2H, t, J = 6·85), 1·54–1·75 (3H, m) |
| (B) | 1·48 (s) | 3·16, 3·18 (dd, J = 5·03, 7·34) | 3·09 (t, J = 7·10) | 0·90 (3H, t, J = 7·59), 0·92 (6H, d, J = 6·59), 1·35–1·53 (2H, m), 1·55–1·80 (3H, m) |
| sec-Bu (A) | 1·49 (s) | 3·13 (t, J = 6·93) | 2·84 (d, J = 5·77) 2·88 (d, J = 4·61) | 0·85–0·95 (9H, m), 1·10–1·75 (5H, m) |
| (B) | 1·48 (s) | 3·16 (t, J = 6·52) 3·19 (t, J = 6·44) | 2·92 (d, J = 5·45) 2·98 (d, J = 4·45) | 0·85–0·95 (9H, m)), 1·10–1·75 (5H, m) |
| $(CH_2)_2$– | 1·48 (s) | 3·10 (t, J = 6·68) | 3·00 (t, J = 6·68) | 0·90, 0·92(3H, each t, J = 7·43, 7·51), |
| $CO_2Na$ (M) | 1·49 (s) | 3·18, 3·20 (dd, J = 5·20, 76·18) | 3·04 (t, J = 6·52) | 1·60–1·90 (4H, c), 2·30–2·50(2H, c) |
| $(CH_2)_3$– $CO_2Na$ (M) | 1·48 (s) 1·49 (s) | 2·96–3·22 (2H, m) | | 0·90, 0·92 (3H, each t, J = 6·85, 6·77), 1·40–1·80 (6H, c), 2·14–2·24 (2H, c) |

the calculated yield presuming consumption of reducing agent. The fractions collected at these two peaks were transferred to the freeze-drying unit, but the fraction eluting from 54·8 to 58·0 min was not collected. Thus the combined synthetic yield of both isomers of 6 was 72·5%. A variety of substituted N-(carboxyalkyl)amino-acid trit-butyl ester derivatives (90 compounds) were automatically synthesized in a similar manner to that described above, and the yields with the elemental analysis are presented in table 2. The $^1$H NMR data are shown in tables 3 to 11. Since no significant influence from the amino-acid substituents was found, the addition rate of sodium cyanoborohydride was computed using the substituent constants of the 2-keto acids. However, the forward rate constants for glyoxylic acid and pyruvic acid were sufficiently large, in these case, for the reducing agent to be added to the reaction mixture in an uncontrolled process.

*Configurational assignment of the products*

The natural L-amino-acids, except for those containing sulphur (cysteine and methionine), have S-configuration on the chiral centre. Thus the reaction of an L-amino-acid tert-butyl ester with a 2-keto acid generally gives two diastereoisomers, [S,R]- and [S,S]-isomers. All of the diastereoisomers derived from the 2-keto acids, except those from glyoxylic acid, pyruvic acid and 2-keto dicarboxylic acid, were separated from each other in the purification unit of the synthesis apparatus. Subsequently, the configurations of the newly formed chiral centres were determined. As a typical example, each diastereoisomer of 6 was derivatized into its N-(carboxy-3-methyl)-butyl-L-leucine disodium salt (7) having symmetrical structure. The first eluted peak (6-a) of 6, converted into the corresponding disodium salt (7-a) by deblocking with trifluoroacetic acid and purified on a Sephadex LH-20 column, gave the meso-compound having no $[\alpha]_D$ value, whereas the disodium salt (7-b) derived from the second eluted peak (6-b) showed a positive $[\alpha]_D$ value (+39·2°). Thus the configuration of the newly formed chiral centre for 6-a was determined as R, and that of 6-b as S.

Typical CD and $[\alpha]_D$ of substituted N-(carboxyalkyl)-

*Table 4. NMR spectra of substituted N-(carboxyalkyl)norvaline tert-butyl esters.*

| R | $Bu^t$ | –CH– | –NHCHR | Other protons |
|---|---|---|---|---|
| H | 1·49 (s) | 3·26 (t, J = 6·60) | 3·16, 3·17<br>(2H, dd, J = 16·5, 18·5) | 0·92 (3H, t, J = 7·34), 1·25–1·43(2H, m),<br>1·59–1·74 (2H, m) |
| Me (M) | 1·48 (s)<br>1·49 (s) | 3·21 (t, J = 6·76)<br>3·26 (t, J = 6·52) | 3·10, 3·11<br>(each q, J = 6·93) | 0·92 (3H, t, J = 7·26), 1·21, 1·22 (3H, each d,<br>J = 6·93), 1·26–1·44 (2H, c), 1·56–1·83 (2H, c) |
| Et (A) | 1·49 (s) | 3·17 (t, J = 6·85) | 2·93, 2·96<br>(dd, J = 5·78, 7·43) | 0·89 (3H, t, J = 7·43), 0·91 (3H, t, J = 7·26),<br>1·23–1·44 (2H, m), 1·50–1·73 (4H, m) |
| Et (B) | 1·48 (s) | 3·23 (t, J = 6·36) | 3·01 (t, J = 6·35) | 0·89 (3H, t, J = 7·59), 0·91 (3H, t, J = 7·25), 1·21 1.44<br>(2H, m), 1·51–1·72 (4H, m) |
| n-Pr (A) | 1·49 (s) | 3·17 (t, J = 6·85) | 3·01 (t, J = 6·60) | 0·912 (3H, t, J = 7·34), 0·905 (3H, t, J = 7·26), 1·21–<br>1·42 (4H, m), 1·50–1·73 (4H, m) |
| n-Pr (B) | 1·48 (s) | 3·23 (t, J = 6·36) | 3·06 (t, J = 6·44) | 0·907 (3H, t, J = 7·26), 0·912 (3H, t, J = 7·25), 1·21–<br>1·43 (4H, m), 1·50–1·72 (4H, m) |
| i-Pr (A) | 1·49 (s) | 3·12 (t, J = 7·01) | 2·75 (d, J = 6·10) | 0·91 (3H, t, J = 7·25), 0·92, 0·95 (6H, each d, J = 6·92),<br>1·24–1·44 (2H, m), 1·53–1·75 (2H, m), 1·75–1·89 (1H, ) |
| i-Pr (B) | 1·48 (s) | 3·18 (t, J = 6·35) | 2·84 (d, J = 5·61) | 0·91 (3H, t, J = 7.26), 0·91, 0·93 (6H, each d, J = 6·93),<br>1·20–1·45 (2H, m), 1·58–1·72 (2H, m), 1·80–1·94<br>(1H, m) |
| n-Bu (A) | 1·49 (s) | 3·18 (t, J = 6·85) | 3·01 (t, J = 6·60) | 0·88 (3H, t, J = 6·93), 0·91 (3H, t, J = 7·25), 1·23–1·42<br>(6H, m), 1·45–1·75 (4H, m) |
| n-Bu (B) | 1·48 (s) | 3·22 (t, J = 6·36) | 3·04 (t, J = 6·52) | 0·89 (3H, t, J = 6·77), 0·91 (3H, t, J = 7·26), 1·20–1·43<br>(6H, m), 1·50–1·72 (4H, m) |
| i-Bu (A) | 1·49 (s) | 3·15 (t, J = 6·85) | 3·04 (t, J = 7·10) | 0·91 (3H, t, J = 7·26), 0·91, 0·92 (6H, each d, J = 6·59),<br>1·25–1·45 (2H, m), 1·39 (2H, t-like, J = 7·10), 1·55–1·75<br>(3H, m) |
| i-Bu (B) | 1·48 (s) | 3·21 (t, J = 6·35) | 3·09 (t, J = 7·01) | 0·91 (3H, t, J = 7·26), 0·92 (6H, d, J = 6·59), 1·20–1·50<br>(4H, m), 1·55–1·72 (3H, m) |
| sec-Bu (A) | 1·50 (s) | 3·03 (t, J = 7·01) | 2·84 (d, J = 5·77)<br>2·89 (d, J = 4·61) | 0·90 (3H, t, J = 7·10), 0·91 (3H, d, J = 7·26),<br>0·93 (3H, t, J = 7·34), 1·10–1·30 (1H, m), 1·35–1·80<br>(6H, m) |
| sec-Bu (B) | 1·48 (s) | 3·11, 3·14<br>dd, J = 4·95, 7·42)<br>3·14, 3·16<br>(dd, J = 5·10, 6·76) | 2·92 (d, J = 5·28)<br>2·98 (d, J = 4·62) | 0·85–0·95 (9H, m), 1·10–1·27 (1H, m),<br>1·35–1·82 (6H, m) |
| $(CH_2)_2$–<br>\|<br>$CO_2Na$ (M) | 1·475 (s)<br>1·489 (s) | 3·17 (t, J = 6·85)<br>3·23 (t, J = 6·44) | 2·99 (t, J = 6·68)<br>3·03 (t, J = 6·43) | 0·91 (3H, t, J = 7·34), 1·25–1·43 (2H, c),<br>1·50–1·88 (4H, c), 2·13–2·27 (2H, c) |
| $(CH_2)_3$–<br>\|<br>$CO_2Na$ (A) | 1·489 (s) | 3·17 (t, J = 6·85) | 2·99<br>(undefined t) | 0·91 (3H, t, J = 7·26), 1·25–1·45 (2H, m),<br>1·50–1·75 (6H, m), 2·19 (2H, undefined t) |
| $(CH_2)_3$–<br>\|<br>$CO_2Na$ (B) | 1·490 (s) | 3·17 (t, J = 6·85) | 3·00<br>(undefined t) | 0·91 (3H, t, J = 7·26), 1·25–1·45 (2H, m),<br>1·50–1·75 (6H, m), 2·19 (2H, undefined t) |

amino-acid tert-butyl esters are shown in table 12. All of the first eluted products (A) and the second eluted products (B) showed negative Cotton effects at 227–237 nm and positive Cotton effects at 220–225 nm in water, respectively.

In the $^1H$ NMR spectra of substituted N-(carboxyalkyl)-amino-acid tert-butyl ester, the chemical shift of the tert-butyl ester group (1·48–1·50 ppm) in the A-isomers was found to be generally at lower field (ca. 0·01 ppm) than in the B-isomers. The chemical shifts of the methine protons on the two chiral centres of the A-isomers (around 2·9–3·6 and 2·7–3·2 ppm) were observed at higher field (0·05–0·1 ppm) than those of the B-isomers.

### Conclusion

The reaction control equations incorporated in a computer program have been applied to the synthesis of substituted N-(carboxyalkyl)amino-acids, and all synthetic processes were automatically performed from the mixing of reactants to the isolation of the products as powders. The automated apparatus was run continuously for 24 hours, giving an average rate of synthesis of three compounds per day. The apparatus extremely valuable for synthesizing many derivatives of a particular compound. Even if the chemical yields are low under the optimum conditions. It is still possible to obtain a sufficient amount of the desired product by repeating the reaction. Moreover, it is possible to greatly reduce the labour-intensive nature of the many syntheses which are a necessary part of pharmaceutical research.

### Experimental

Infra-red and ultraviolet spectra were measured on Hitachi 260-10 and Hitachi 557 double wavelength spectrophotometers, respectively. Optical rotations

*Table 5. NMR spectra of substituted N-(carboxyalkyl)valine tert-butyl esters.*

| R | $Bu^t$ | –CH– | –NHCHR | Other protons |
|---|---|---|---|---|
| H | 1·495 (s) | 3·04 (d, J = 5·77) | 3·07, 3·14<br>(2H, dd, J = 16·4, 19·9) | 0·97 (6H, t, J = 6·85), 1·99 (1H, sex, J = 6·67) |
| Me (M) | 1·484 (s)<br>1·496 (s) | 2·94 (d, J = 6·60) | 3·00–3·12 (c) | 0·90–1·03 (6H, c), 1·22 (3H, d, J = 6·92),<br>1·90–2·05 (1H, c) |
| Et (M) | 1·483 (s)<br>1·499 (s) | 2·89 (d, J = 6·76)<br>3·04 (d, J = 5·80) | 2·87, 2·90<br>(dd, J = 5·61, 7·75)<br>2.96 (t, J = 6·27) | 0·84–1·02 (9H, c), 1·96 (1H, sep, J = 6·73) |
| n-Pr (M) | 1·483 (s)<br>1·500 (s) | 2·87 (d, J = 6·93)<br>3·03 (d, J = 5·61) | 2·95 (t, J = 6·85)<br>3·02 (t, J = 6·02) | 0·86–1·01 (9H, c), 1·2–1·4 (2H, c), 1·4–1·63<br>(2H, c), 1·95 (1H, sep, J = 6·64) |
| i-Pr (M) | 1·480 (s)<br>1·500 (s) | 2·67 (d, J = 6·26)<br>2·77 (d, J = 5·61) | 2·78 (d, J = 7·43)<br>2·98 (d, J = 5·77) | 0·85–1·03 (12H, c), 1·70–2·03 (2H, c) |
| n-Bu (A) | 1·501 (s) | 2·87 (d, J = 6·93) | 2·92, 2·95<br>(dd, J = 6·11, 7·43) | 0·89 (3H, t, J = 6·93), 0·93, 0·97 (6H, each d, J = 6·77), 1·23–1·40 (4H, m), 1·47–1·65 (2H, m), 1·92 (1H, sex, J = 6·93) |
| n-Bu (B) | 1·483 (s) | 3·03 (d, J = 5·61) | 2·99 (t, J = 6·35) | 0·89 (3H, t, J = 6·93), 0·94, 0·98 (6H, each d, J = 6·76, 6·92), 1·22–1·38 (4H, m), 1·50–1·65 (2H, m), 1·97 (1H, sex, J = 6·54) |
| i-Bu (A) | 1·505 (s) | 2·86 (d, J = 6·93) | 2·99 (t, J = 7·10) | 0.84–1·01 (12H, m), 1·40 (2H, t, J = 6·85), 1·54–1·70 (1H, m), 1·94 (1H, sex, J = 6·76) |
| i-Bu (B) | 1·485 (s) | 3·03 (d, J = 5·45) | 3·03 (t, J = 6·43) | 0·91, 0·92, 0·93, 0·98 (each 3H, each d, J = 6·43, 6·59, 6·93, 6·92), 1·37–1·50 (2H, m), 1·65 (1H, sep, J = 6·77), 1·96 (1H, sex, 6·76) |
| sec-Bu (M) | 1·476 (s)<br>1·481 (s)<br>1·499 (s) | 2·70–3·00 | | 0·80–1·03 (12H, c), 1·0–1.3 (1H, c), 1·30–1·75 (2H, c), 1·93 (1H, undefined sex) |
| $(CH_2)_2$–<br>\|<br>$CO_2Na$ (M) | 1·480 (s)<br>1·500 (s) | 2·89 (d, J = 6·76)<br>4·04 (d, J = 5·44) | 2·92, 2·95 (dd, J = 6·44, 7·43), 2·99 (t, J = 6·43) | 0·93, 0·95 (3H, each, d, J = 6·77), 0·976, 0·982 (3H, each d, J = 6·93), 1·7–1·9 (2H, c), 1·96 (1H, undefined sep), 2·1–2·3 (2H, c) |
| $(CH_2)_3$–<br>\|<br>$CO_aNa$ (M) | 1·481 (s)<br>1·500 (s) | 2·88 (d, J = 6·76)<br>3·04 (d, J = 5·44) | 2·90–3·07 (c) | 0·93, 0·94, 0·97, 0·98 (6H, each d, J = 6·93), 1·50–1·65 (4H, c), 1·95 (1H, sep, J = 6·92), ca. 2·19 (2H, undefined br. t) |

*Table 6. NMR spectra of substituted N-(carboxyalkyl)norleucine tert-butyl ester.*

| R | $Bu^t$ | –CH– | –NHCHR | Other protons |
|---|---|---|---|---|
| H | 1·501 (s) | 3·25 (t, J = 6·52) | 3.11, 3.17 (2H, dd, J = 16·5, 17·8) | 0·90 (3H, t, J = 6·77), 1·23–1·42, (4H, m), 1·60–1·77 (2H, m) |
| Me (M) | 1·478 (s)<br>1·486 (s) | 3·20 (t, J = 6·77)<br>3·24, 3·27 (dd, J = 5·78, 7·26) | 3·10 (q, J = 7·09)<br>3·11 (q, J = 6·93) | 0·89 (3H, t, J = 6·77), 1·21, 1·22 (3H, each d, J = 6·93, 7·09), 1·22–1·39 (4H, c), 1·57–1·75 (2H, c) |
| Et (M) | 1·478 (s)<br>1·489 (s) | 3·16 (t, J = 6·85)<br>3·21 (t, J = 6·35) | 2·93,. 2·96 (dd, J = 5·86, 7·18), 3·00 (t, J = 6.27) | 0·89 (6H, t, J = 7·51), 1·2–1·4 (2H, c), 1·5–1·74 (6H, c) |
| n-Pr (M) | 1·477 (s)<br>1·490 (s) | 3·15 (t, J = 6·77)<br>3·21 (t, J = 6·35) | 3·00, 3·05<br>(each t, J = 6·60) | 0·83–0·96 (6H, c), 1·2–1·4 (6H, c), 1·41–1·75 (4H, c) |
| i-Pr (M) | 1·476 (s)<br>1·491 (s) | 3·09 (t, J = 7·02)<br>3·17 (t, J = 6·36) | 2·74 (d, J = 5·93)<br>2·83 (d, J = 5·61) | 0·82–1·00 (9H, c), 1·2–1·4 (4H, c), 1·55–1·75 (2H, c), 1·75–1·94 (1H, c) |
| n-Bu (M) | 1·477 (s)<br>1·490 (s) | 3·15 (t, J = 6·85)<br>3·21 (t, J = 6·27) | 2·99 (t, J = 6·52)<br>3·03 (t, J = 6·27) | 0·81–0·95 (6H, c), 1·2–1.4 (8H, c), 1·45–1·75 (4H, c) |
| i-Bu (M) | 1·480 (s)<br>1·495 (s) | 3·14 (t, J = 6·93)<br>3·21 (t, J = 6·35) | 3·04 (t, J = 6·19)<br>3·09 (t, J = 6·60) | 0·82–0·96 (9H, c), 1·2–1·75 (9H, c) |
| sec-Bu (M) | 1·475 (s)<br>1·489 (s) | 3·02–3·22 (s) | 2·82 (d, J = 5·77)<br>2·87 (d, J = 4·62)<br>2·90 (d, J = 5·28)<br>2·97 (d, J = 4·29) | 0·82–0·97 (9H, c), 1·10–1·75 (9H, c) |
| $(CH_2)_2$–<br>\|<br>$CO_2Na$ (M) | 1·476 (s)<br>1·490 (s) | 3·16 (t, J = 6·77)<br>3·23 (t, J = 6·36) | 2·99 (t, J = 6·60)<br>3·04 (t, J = 6·19) | 0·89 (3H, t, J = 7·10), 1·22–1·40 (4H, c), 1·56–1·88 (4H, c), 2·14–2·26 (2H, c) |
| $(CH_2)_3$–<br>\|<br>$CO_2Na$ (M) | 1·476 (s)<br>1·489 (s) | 3·15 (t, J = 6·85)<br>3·22 (t, J = 6·36) | 2·94–3·09 (c) | 0·89 (3H, t, J = 6·69), 1·2–1·4 (4H, c), 1·50–1·75 (6H, c), 2·19 (2H, c) |

*Table 7. NMR spectra of substituted N-(carboxyalkyl)leucine tert-butyl esters.*

| R | $Bu^t$ | –CH– | –NHCHR | Other protons |
|---|---|---|---|---|
| H | 1·484 (s) | 3·25, 3·28 (dd, J = 6·43, 7·92) | 3·12 (2H, br.s) | 0·92 (3H, d, J = 6·27), 0·94 (3H, d, J = 6·43), 1·40–1·75 (3H, m) |
| Me (M) | 1·475 (s)<br>1·481 (s) | 3·22–3·32 (c) | 3·02–3·16 (c) | 0·85–0·95 (6H, c), 1·21, 1·22 (3H, each d, J = 6·93), 1·30–1·70 (3H, c) |
| Et (M) | 1·474 (s)<br>1·486 (s) | 3·18–3·29 (c) | 2·92, 2·94 (dd, J = 5·86, 7·34), 3·01 (t, J = 6·27) | 0·83–0·97 (9H, c), 1·4–1·7 (5H, c) |
| n-Pr (M) | 1·473 (s)<br>1·487 (s) | 3·17–3·28 (c) | 2·99 (t, J = 6·60)<br>3·05 (t, J = 6·43) | 0·85–0·98 (12H, c), 1·4–1·7 (3H, c), 1·70–1·93 (1H, c) |
| i-Pr (M) | 1·473 (s)<br>1·487 (s) | 3·13–·24 (c) | 2·73 (d, J = 6·10)<br>2·84 (d, J = 5·45) | 0·85–0·97 (9H, c), 1·23–1·40 (1H, c), 1·4–1·7 (1H, c) |
| n-Bu (A) | 1·489 (s) | 3·23 (t, J = 7·02) | 2·98 (t, J = 6·88) | 0·83–0·97 (9H, undefined m), 1·23–1·38 (4H, m), 1·4–1·7 (5H, m) |
| n-Bu (B) | 1·475 (s) | 3·23, 3·26 (dd, J = 5·61, 8·09) | 3·04 (t, J = 6·35) | 0·89 (3H, t, J = 6·76), 0·92 (6H, d, J = 6·43), 1·23–1·38 (4H, m), 1·38–1·70 (5H, m) |
| i-Bu (A) | 1·492 (s) | 3·23 (t, J = 7·10) | 3·04 (t, J = 7·10) | 0·83–0·98 (12H, undefined m), 1·39 (4H, t, J = 6·93), 1·5–1·7 (2H, m) |
| i-Bu (B) | 1·476 (s) | 3·23, 3·26 (dd, J = 5·62, 8·09) | 3·08 (t, J = 7·10) | 0·92 (12H, d, J = 6·27), 1·35–1·72 (6H, m) |
| sec-Bu (M) | 1·473 (s)<br>1·486 (s) | 3·11–3·25 (c) | 2·81 (d, J = 5·93)<br>2·86 (d, J = 4·62)<br>2·92 (d, J = 5·12)<br>2·99 (d, J = 4·29) | 0·82–0·97 (12H, c), 1·08–1·28 (1H, c), 1·3–1·75 (5H, c) |
| $(CH_2)_2$-<br>$CO_2Na$ (M) | 1·472 (s)<br>1·486 (s) | 3·23 (t, J = 6·93)<br>·26 (t, J = 6·68) | 2·98 (t, J = 6·77)<br>3·04 (t, J = 6·35) | 0·87–0·96 (6H, c), 1·4–1·7 (3H, c), 1·7–1·9 (2H, c), 2·12–2·27 (2H, c) |
| $(CH_2)_3$–<br>$CO_2Na$ (M) | 1·473 (s)<br>1·487 (s) | 3·23 (t, J = 6·93)<br>3·25 (t, t-like) | 2·98, 305 (each t-like) | 0·86–0·96 (6H, c), 1·4–1·7 (7H, c), 2·19 (2H, t, J = 6·59) |

*Table 8. NMR spectra of substituted N-(carboxyalkyl)isoleucine tert-butyl ester.*

| R | $Bu^t$ | –CH– | –NHCHR | Other protons |
|---|---|---|---|---|
| H | 1·49 (s) | 3·17 (d, J = 5·28) | 3·08, 3·15 (dd, J = 16·5, 18·3) | 0·92 (3H, t, J = 6·68), 0·93 (3H, d, J = 6·11), 1·17–1·57 (2H, m) |
| Me (A) | 1.49 (s) | 3·08 (d, J = 6·11) | 3·07 (q, J = 6·92) | 0·92 (3H, t, J = 7·43), 0·92 (3H, d J = 7·09), 1·22 (3H, d, J = 6·92), 1·34–1·83 (3H, m) |
| Me (B) | 1·48 (s) | 3·24 (d, J = 4·95) | 3·10 (q, J = 6·93) | 0·86–0·98 (6H, c), 1·23 (3H, d, J = 6·93), 1·38–1·83 (3H, c) |
| Et (A) | 1·50 (s) | 3·03 (d, J = 6·44) | 2·88, 2·91 (dd, J = 5·69, 7·67) | 0·89 (3H, t, J = 7·43), 0·90 (3H, t, J = 7·01), 0·91 (3H, d, J = 6·92), 1·16–1·80 (5H, c) |
| Et (B) | 1·48 (s) | 3·16 (d, J = 5·28) | 2·95 (d, J = 6·27) | 0·89 (3H, t, J = 7·26), 0·92 (3H, t, J = 7·26), 0·92 (3H, d, J = 6·60), 1·19–1·65 (5H, c) |
| n-Pr (A) | 1·50 (s) | 3·01 (d, J = 6·60) | 2·95, 2·97 (dd, J = 6·19, 7·18) | 0·90 (3H, t, J = 6·68), 0·91 (3H, t, J = 7·18), 0·91 (3H, d, J = 6·26), 1·71–1·82 (7H, c) |
| n-Pr (B) | 1·48 (s) | 3·16 (d, J = 5·11) | 2·99 (t, J = 6·36) | 0·91 (3H, t, J = 7·26), 0·91 (3H, d, J = 6·59), 0·92 (3H, t, J = 6·82), 1·18–1·80 (7H, c) |
| i-Pr (A) | 1·50 (s) | 2·94 (d, J = 7·26) | 2·69 (d, J = 6·27) | 0·90 (3H, t, J = 7·34), 0·90, 0·91 (6H, each d, J = 6·92), 1·26–1·88 (4H, c) |
| i-Pr (B) | 1·48 (s) | 3·12 (d, J = 5·11) | 2·76 (d, J = 5·77) | 0·92 (12H, t-like, J = 6·85), 1·20–1·35 (1H, m), 1·65–1·93 (3H, m) |
| n-Bu (A) | 1·50 (s) | 3·01 (d, J = 6·44) | 2·93, 2·96 (dd, J = 6·02, 7·51) | 0·88 (3H, t, J = 6·85), 0·90 (3H, t, J = 7·26), 0·91 (3H, d, J = 6·42), 1·20–1·80 (9H, c) |
| n-Bu (B) | 1·48 (s) | 3·16 (d, J = 5·11) | 2·99 (t, J = 6·35) | 0·89 (3H, t, J = 6·76), 0·91 (3H, d, J = 6·76), (0·92 (3H, t, J = 7·26), 1·20–1·80 (9H, c) |
| i-Bu (A) | 1·50 (s) | 3·00 (d, J = 6·60) | 3·00 (t, J = 7·18) | 0·85–0·95 (12H, m), 1·15–1·35 (1H, m), 1·40 (2H, t-like, J = 6·77), 1·45–1·85 (3H, m) |
| i-Bu (B) | 1·48 (s) | 3·16 (d, J = 5·11) | 3·03 (t, J = 7·01) | 0·89–0·95 (12H, m), 1·21–1·37 (1H, m), 1·42 (2H, t, J = 6·93), 1·40–1·75 (3H, m) |
| sec-Bu (A) | 1·50 (s) | 2·96 (d, J = 7·26) | 2·80 (d, J = 5.94)<br>2·82 (d, J = 5·11) | 0·85–0·95 (9H, m), 1·08–1·34 (2H, m), 1·36–1·68 (4H, m) |
| sec-Bu (B) | 1·47 (s) | 3·09 (d, J = 5·28)<br>3·11 (d, J = 5·11) | 2·85 (d, J = 5·44)<br>2·92 (d, J = 4·46) | 0·84–0·96 (9H, m), 1·10–1·36 (2H, m), 1·36–1·80 (4H, m) |

*Table 8 (continued).*

| R | $Bu^t$ | –CH– | –NHCHR | Other protons |
|---|---|---|---|---|
| $(CH_2)_2$–$CO_2Na$ (A) | 1·50 (s) | 3·03 (d, J = 6·43) | 2·94, 2·96 (dd, J = 6·11, 7·43) | 0·90 (3H, t, J = 6·92), 0·91 (3H, d, J = 7·26), 1·15–1·35 (1H, m), 1·70–1·90 (4H, m), 2·10–2·25 (2H, m) |
| $(CH_2)_2$–$CO_2Na$ (B) | 1·48 (s) | 3·17 (d, J = 4·95) | 2·99 (t, J = 6·44) | 0·92 (3H, t, J = 7·26), 0·92 (3H, d, J = 6·93), 1·15–1·30 (1H, m), 1·40–1·65 (5H, m), 2·15–2·30 (2H, m) |
| $(CH_2)_3$–$CO_2Na$ (A) | 1·50 (s) | 3·02 (d, J = 6·43) | 2·95 (t, J = 6·10) | 0·90 (3H, t, J = 7·26), 0·91 (3H, d, J = 6·60), 1·15–1·30 (1H, m), 1·40–1·65 (5H, m), 1·65–1·80 (1H, m), 2·19 (2H, t, J = 7·09) |
| $(CH_2)_3$–$CO_2Na$ (B) | 1·48 (s) | 3·17 (d, J = 4·95) | 3·00 (undefined t) | 0·91 (3H, d, J = 6·76), 0·92 (3H, t, J = 7·26), 1·15–1·45 (1H, m), 1·40–1·80 (6H, m), 2·19 (2H, undefined t) |

*Table 9. NMR spectra of substituted N-(carboxyalkyl)serine-O-tert-butyl ester.*

| R | $Bu^t$ | –CH– | –NHCHR | Other protons |
|---|---|---|---|---|
| H | 1·490 (s) | 3·38 (t, J = 6·02) | 3·14, 3·22 (2H, dd, J = 16·5, 20·8) | 1·21 (9H, s), 3·61, 3·63 (2H, each d, J = 6·02) |
| Me (M) | 1·487 (s)<br>1·491 (s) | 3·38 (t, J = 6·60) | 3·16 (q, J = 6·93)<br>3·19 (q, J = 7·09) | 1·206, 1·214 (9H, each s), 1·23 (3H, d, J = 7·00), 3·52–3·69 (2H, c) |
| Et (M) | 1·486 (s)<br>1·494 (s) | 3·34 (t, J = 6·60) | 3·01, 3·03 (dd, J = 5·86, 7·34), 3·08 (t,. J = 6·36) | 0·88, 0·89 (3H, each t, J = 7·51), 1·20–1·21 (9H, each s), 1·45–1·75 (2H, c), 3·52–3·69 (2H, c) |
| n-Pr (M) | 1·485 (s)<br>1·495 (s) | 3·33, 3·35 (dd, J = 5·20, 6·85) | 3·08 (t, J = 6·60)<br>3·12 (t, J = 6·35) | 0·91 (3H, t, J = 7·26), 1·2–1·6 (4H, c), 1·206, 1·213 (9H, each s), 3·51–3·54 (2H, c) |
| i-Pr (M) | 1·484 (s)<br>1·495 (s) | 3·28 (t, J = 6·60)<br>3·28, 3·31 (dd, J = 4·95, 10·06) | 2·82 (d, J = 6·10)<br>2·90 (d, J = 5·60) | 0·92, 0·95 (3H, each d, J = 6·93, 6·76), 1·207, 1·213 (9H, each s), 1·85 (1H, sep, J = 6·76), 3·53–3·68 (2H, c) |
| n-Bu (A) | 1·495 (s) | 3·33 (t, J = 6·68) | 3·06 (t, J = 6·60) | 0·88 (3H, t, J = 6·77), 1·21 (9H, s), 1·23–1·36 (4H, m), 1·47–1·65 (2H, m), 3·52–3·65 (2H, m) |
| n-Bu (B) | 1·486 (s) | 3.33, 3.36 (dd, J = 5·44, 6·76) | 3·12 (t, J = 6·52) | 0·89 (3H, t, J = 6·68), 1·21 (9H, s), 1·2–1·4 (4H, m), 1·5–1·64 (2H, m), 3·52–3·68 (2H, m) |
| i-Bu (A) | 1·498 (s) | 3·32 (t, J = 6·68) | 3·11 (t, J = 7·09) | 0·91, 0·92 (6H, each d, J = 6·43, 6·60), 1·21 (9H, s), 1·41 (2H, t-like, J = 6·77), 1·64–1·70 (1H, m), 3·53–3·65 (2H, m) |
| i-Bu (B) | 1·488 (s) | 3·33, 3·35 (dd, J = 5·11, 6·76) | 3·15 (t, J = 7·01) | 0·92 (6H, d, J = 6·60), 1·21 (9H, s), 1·34–1·53 (2H, m), 1·53–1·68 (1H, m), 3·52–3·69 (2H, m) |
| sec-Bu (M) | 1·485 (s)<br>1·495 (s) | 3·24–3·34 (c) | 2·89–3·08 (c) | 0·82–0·97 (6H, c), 1·207, 1·214 (9H, each s), 1·40–1·75 (3H, c), 2·89–3·08 (1H, c), 3·53–3·68 (2H, c) |
| $(CH_2)_2$–$CO_2Na$ (M) | 1·184 (s)<br>1·495 (s) | 3·3–3·4 (c) | 3·07 (t, J = 6·68)<br>3·10 (t, J = 6·52) | 1·207, 1·213 (9H, each s), 1·73–1·89 (2H, c), 2·13–2·27 (2H, c), 3·52–3·70 (2H, c) |
| $(CH_2)_3$–$CO_2Na$ (M) | 1·484 (s)<br>1·495 (s) | 3·29–3·38 (c) | 3·01–3·15 (c) | 1·205, 1·212 (9H, each s), ca. 1·51 (4H, br. s), ca. 2·20 (2H, br. t), 3·52–3·69 (2H, c) |

*Table 10. NMR spectra of substituted N-(carboxyalkyl)threonine-O-tert-butyl ester.*

| R | $Bu^t$ | –CH– | –NHCHR | Other protons |
|---|---|---|---|---|
| H | 1·50 (s) | 3·21 (d, J = 6·93) | 3·05, 3·17 (2H, dd, J = 16·4, 32·4) | 1·19 (3H, d, J = 6·42), 1·27 (9H, s), 3·96 (1H, quin, J = 6·44) |
| Me (M) | 1·492 (s)<br>1·499 (s) | 3·11 (d, J = 7·58)<br>3·34 (d, J = 5·77) | 3·00–3·14 (c) | 1·12–1·30 (6H, c), 1·25, 1·27 (9H, each s), 3·83–4·03 (1H, c) |
| Et (A) | 1·50 (s) | 3·08 (d, J = 7·42) | 2·89, 2·92 (dd, J = 5·28, 7·92) | 0·88, (3H, t, J = 7·51), 1·17 (3H, d, J = 6·27), 1·27 (9H, s), 1·40–1·75 (2H, m), 3·87–3·92 (1H, m) |
| Et (B) | 1·49 (s) | 3·29 (d, J = 5·94) | 3·00 (t, J = 6·03) | 0·87 (3H, t, J = 7·51), 1·19 (3H, d, J = 6·44), 1·26 (9H, s), 1·54–1·74 (2H, m), 3·98 (1H, quin, J = 6·27) |
| n-Pr (M) | 1·49 (s)<br>1·50 (s) | 3·07 (d, J = 7·59)<br>3·29 (d, J = 5·94) | 2·94, 2·97 (dd, J = 5·77, 7·42), 3·03 (t, J = 6·60) | 0·88, 0·93 (3H, each t, J = 7·26), 1·16, 1·19 (6H, each d, J = 4·76, 4·95), 1·26, 1·27 (9H, each s), 1·30–1·70 (4H, c), 3·89, 3·97 (1H, each quin, J = 6·3) |

*Table 10 (continued).*

| R | Bu$^t$ | –CH– | –NHCHR | Other protons |
|---|---|---|---|---|
| i-Pr (M) | 1·49 (s)<br>1·50 (s) | 3·05 (d, J = 7·09)<br>3·26 (d, J = 5·78) | 2·71 (d, J = 6·11)<br>2·80 (d, J = 5·61) | 0·90 (3H, d, J = 6·93), 0·96 (3H, d, J = 6·60), 1·17, 1·19 (3H, each d, J = 6·10), 1.25, 1·27 (9H, each s), 1·7–2·0 (1H, c), 3·92, 4·00 (1H, each quin) |
| n-Bu (M) | 1·49 (s)<br>1·50 (s) | 3·07 (d, J = 7·42)<br>3·29 (d, J = 6·11) | 3·02 (t, J = 6·19), 2·93, 2·96 (dd, J = 5·77, 7·59) | 0·83–0·93 (6H, c), 1·26, 1·27 (9H, each s), 1·14–1·65 (6H, c), 3·89, 3·97 (1H, each quin, J = 6·85, 6·35) |
| i-Bu (M) | 1·49 (s)<br>1·51 (s) | 3·06 (d, J = 7·42)<br>3·30 (d, J = 5·94) | 2·93–3·06 (c) | 0·85–0·97 (9H, c), 1·25, 1·26 (9H, each s), 1·12–1·25 (2H, c), 3·89, 3·96 (1H, each quin, J = 6·76, 6·27) |
| sec-Bu (M) | 1·48 (s)<br>1·49 (s)<br>1·50 (s) | 3·03 (d, J = 8·08)<br>3·06 (d, J = 7·43)<br>3·24 (d, J = 5·77)<br>3·25 (d, J = 5.94) | 2·80 (d, J = 5·77)<br>2·83 (d, J = 4·95)<br>2·90 (d, J = 5·27)<br>2·95 (d, J = 4·45) | 0·82–0·97 (6H, c), 1·1–1·3 (2H, c), 1·25, 1·26, 1·27 (9H, each s), 1·32–1·74 (1H, c), 3·84–4·05 (1H, c) |
| $(CH_2)_2$–<br>\|<br>$CO_2Na$ (M) | 1·49 (s)<br>1·50 (s) | 3·09 (d, J = 7·26)<br>3·29 (d, J = 5·61) | 2·94, 2·96 (dd, J = 5·69, 7·67) | 1·17, 1·19 (3H, each d, J = 6·43, 6·44), 1·25, 1·27 (9H, each s), 1·70–1·94 (2H, c), 2·18 (2H, t, J = 7·92), 3·91, 4·00 (1H, quin, 6·68, 6·11) |
| $(CH_2)_3$–<br>\|<br>$CO_2Na$ (M) | 1·49 (s)<br>1·50 (s) | 3·07 (d, J = 7·42)<br>3·29 (d, J = 5·94) | 2·94 (t, J = 6·02)<br>3·03 (t, J = 5·53) | 1·17, 1·19 (3H, each d, J = 6·10, 5·78), 1·25, 1·27 (9H, each s), 1·4–1·7 (4H, c), 2·12–2·24 (2H, undefined t), 3·89, 3·98 (1H, each quin, J = 6·85, 6·19) |

*Table 11. NMR spectra of substituted N-(carboxyalkyl)aspartic acid tert-butyl esters.*

| R | Bu$^t$ | –CH– | –NHCHR | Other protons |
|---|---|---|---|---|
| H | 1·481 (s)<br>1·492 (s) | 3·53 (d, J = 5·12)<br>3.56 (d, J = 5·11) | 3·19, 3·20 (2H, each s) | 2·6–2·8 (2H, m) |
| Me (M) | 1·459 (s), 1·470 (s)<br>1·473 (s), 1·482 (s) | 3·53 (t, J = 5·12)<br>3·56 (t, J = 5·11) | 3·20 (q, J = 6·98)<br>3·23 (q, J = 6·93) | 1·22, 1·23 (3H, each d, J = 6·92), 2·56–2·80 (2H, c) |
| Et (M) | 1·461 (s), 1·468 (s)<br>1·474 (2), 1·484 (s) | 3·46–3·57 (c) | 3·06 (t, J = 6·27)<br>3·10 (t, J = 6·43) | 0·89 (3H, t, J = 7·51), 1·5–1·7 (2H, c), 2·6–2·8 (2H, c) |
| n-Pr (M) | 1·460 (s)<br>1·468 (s)<br>1·474 (s)<br>1·485 (s) | 3·47, 3·49<br>(dd, J = 6·19, 7·51)<br>3·52, 3·55<br>(dd, J = 5·03, 7·84) | 3·11 (t, J = 6·44)<br>3·16 (t, J = 6·52) | 0·91 (3H, t, J = 7·26), 1·2–1·6 (4H, c), 2·6–2·8 (2H, c) |
| i-Pr (M) | 1·462 (s)<br>1·466 (s)<br>1·476 (s)<br>1·486 (s) | 3·41, 3·43<br>(dd, J = 6·11, 7·26)<br>3·48, 3·51<br>(dd, J = 4·86, 7·67) | 2·87 (d, J = 5·61)<br>2·93 (d, J = 5·94) | 0·88–0·96 (6H, c), 1·78–1·93 (1H, c), 2·63–2·78 (2H, c) |
| n-Bu (M) | 1·461 (s)<br>1·469 (s)<br>1·475 (s)<br>1·486 (s) | 3·47, 3·49<br>(dd, J = 6·02, 7·34)<br>3·52, 3·55<br>(dd, J = 5·11, 7·59) | 3·10 (t, J = 6·35)<br>3·14 (t, J = 6·52) | 0·89 (3H, t, J = 6·85), 1·2–2·8 (8H, c) |
| i-Bu (M) | 1·459 (s)<br>1·467 (s)<br>1·476 (s)<br>1·487 (s) | 3·48, 3·51<br>(dd, J = 6·02, 7·34)<br>3·50, 3·58<br>(dd, J = 4·95, 7·59) | 3·13 (t, J = 7·09)<br>3·20 (t, J = 7·01) | 0·92, 0·93 (6H, each d, J = 6·43), 1·55–1·75 (1H, c), 1·3–1·5 (2H, c), 2·6–2·8 (2H, c) |
| sec-Bu (M) | 1·461 (s), 1·466 (s)<br>1·476 (s), 1·484 (s)<br>1·486 (s) | 3.35–3.55 (c) | 2·9–3·1 (c) | 0·80–0·95 (6H, c), 1·1–1·3 (1H, c), 1·5–1·7 (2H, c), 2·60–2·75 (2H, c) |
| $(CH_2)_2$–<br>\|<br>$CO_2Na$ (M) | 1·460 (s), 1·464 (s)<br>1·469 (s), 1·472 (s)<br>1·485 (s) | 3·45–3·60 (c) | 3·05–3·20 (c) | 1·7–1·85 (2H, c), 2·15–2·25 (2H, c), 2·65–2·75 (2H, c) |
| $(CH_2)_3$–<br>\|<br>$CO_2Na$ (M) | 1·459 (s), 1·466 (s)<br>1·472 (s), 1·485 (s) | 3·45–3·60 (c) | 3·15 (br. s) | 1·55 (4H, c), 2.20 (2H, c), 2·6–2·8 (2H, c) |

($[\alpha]_D$) and CD spectra were measured with JASCO DP1-181 and J-20 (Japan Spectroscopic Co., Ltd) spectrophotomers, respectively. $^1$H NMR spectra were measured with a JEOL JNM-GX270 spectrometer using 3-(trimethylsilyl)propionic acid $d_4$-sodium salt as an internal standard. Personal computers were NEC PC-9801 and Fujitsu FM 77AV. The instrument used for HPLC analysis was a Shimadzu LC-5A, equipped with a Nucleosil 5C18 column (4 × 250 mm) and operated as follows: flow rate 0·7 ml/min; detector UV-235 nm; chart speed 2·5 mm/min; mobile phase, a mixture of 0.05M aq-AcONa and MeOH (3 : 7, v/v). N-(Carboxy-3-methyl)-butyl-L-leucine disodium salt (7).

Both isomers (6-a and 6-b) of N-(3-methyl-l-sodioxycarbonyl)-butyl-L-leucine tert-butyl ester, which were

*Table 12. Circular dichroism (CD) and [alpha]$_D$ of substituted N-(carboxyalkyl)amino acid tert-butyl esters.*

| Substituents* | | Retention | $[\alpha]_D$ (24°) | CD |
|---|---|---|---|---|
| $R_1$ | $R_s$ | time† (min) | (c in $H_2O$) | $\lambda_{max}$ nm ($\Delta\varepsilon$ in $H_2O$) |
| i-Pr | H | 3·81 | −13·4 (0·62) | 210 (+0·92) |
| | n-Bu (A) | 5·23 | −23·3 (0·68) | 229 (−0·56) |
| | n-Bu (B) | 7·25 | −14·9 (0·65) | 223 (+0·67) |
| | i-Bu (A) | 5·20 | −27·8 (0·49) | 229 (−0·56) |
| | i-Bu (B) | 7·05 | −12·5 (0·44) | 223 (+0·76) |
| i-Bu | H | 4·34 | −5·7 (0·68) | 210 (+0·81) |
| | n-Bu (A) | 6·09 | −15·1 (0·64) | 234 (−0·16) |
| | n-Bu (B) | 8·91 | −6·6 (0·56) | 221 (+0·58) |
| | i-Bu (A) | 6·05 | −19·3 (0·62) | 237 (−0·11) |
| | i-Bu (B) | 8·99 | −3·9 (0·47) | 223 (+0·63) |
| sec-Bu | H | 4·36 | −3·8 (0·63) | 211 (+1·16) |
| | Me (A) | 4·33 | −24·8 (0·56) | 230 (−0·19) |
| | Me (B) | 4·79 | −15·7 (0·37) | 224 (+1·52), 271 (−0·97) |
| | Et (A) | 4·60 | −26·3 (1·03) | 228 (−0·37) |
| | Et (B) | 5·70 | +0·5 (1·08) | 220 (+1·15) |
| | n-Bu (A) | 6·29 | −19·9 (1·14) | 231 (−0·35) |
| | n-Bu (B) | 8·94 | −6·2 (0·95) | 224 (+1·00) |
| | i-Bu (A) | 6·36 | −24·6 (0·89) | 231 (−0·35) |
| | i-Bu (B) | 8·73 | −3·9 (1·00) | 223 (+0·93) |
| CH–$CH_3$ | H | 4·59 | −9·6 (0·53) | 233 (−0·13) |
| \| | Et (A) | 4·83 | −25·0 (0·60) | 227 (−0·83) |
| $OBu^t$ | Et (B) | 6·03 | +8·0 (0·67) | 222 (+0·45) |

(A) and (B) denote the 1st and 2nd eluted products by an Amberlite XAD-2 column.

† HPLC analytical conditions are described in the experimental part.

obtained by the automated synthesis described in the text, were further purified on a XAD-2 column using 50% methanol as an eluent. The eluted solutions were evaporated to half of their volume under reduced pressure, and then freeze-dried. To the purified powder of 6-a (0·3 g) was added chilled trifluoroacetic acid (5 ml) and the mixture was stirred at room temperature for 2 hours. After evaporation of trifluoroacetic acid under reduced pressure, the residue was dissolved in water (5 ml) and treated with 1 N aq-NaOH to adjust it to pH 11. The resulting solution was charged onto a column of Sephadex LH-20 (35 × 500 mm) and eluted with water to obtain N-(1-carboxy-3-methyl)butyl-L-leucine disodium salt (0·21 g; 7-a) after lyophilization. Found: C, 44·37; H, 7·27; N, 4·43%. Calcd for $C_{12}H_{21}NNa_2O_4.2H_2O$: C, 44·31; H, 7·75; N, 4·31%. $^1H$ NMR (ppm in $D_2O$) [δ]: 0·916 (6H, d, j = 6·43), 0·935 (6H, d, J = 6·43), 1·540 (4H, t, J = 6·93), 1·672 (2H, sextet, J = 6·63), 3.289 (2H, t, J = 7·09). $[\alpha]_D$ 0°(c = 0·43 in $H_2O$, 20°C). IR [v]KBr $cm^{-1}$: 2960, 1619, 1446, 1409 and 1383. In a similar manner, 7-b isomer (0·19 g) of N-(1-carboxy-3-mehtyl)-butyl-L-leucine disodium salt was obtained starting from 6-b (0·3 g). Found: C, 43·88; H, 7·45; N, 4.08%. Calcd for $C_{12}H_{21}NNa_2O_4.2H_2O$: C, 44·31; H, 7·75; N, 4·31%. $^1H$ NMR (ppm in $D_2O$) [v]: 0·938 (6H, d, J = 6·44), 0·947 (6H, D, J = 6·27), 1·50–1·78 (6H, complex m), 3·392 (2H, t, J = 6·85). $[\alpha]_D$ +39·2° (c = 0·47 in $H_2O$, 20°). IR $[v]^{KBr}$ $cm^{-1}$: 2958, 1629, 1452 and 1411.

### References

1. Hayashi, N., Sugawara, T., Shintani, M. and Katao, S., *Journal Automatic Chemistry,* **11** (1989), 212.
2. Hayashi, N., *Farumashia,* **26** (1900), 931.
3. Hayashi, N. and Sugawara, T., *Chemical Letters* (1988), 1613.
4. Hayashi, N. and Sugawara, T., *Tetra. Computer Methods,* **1** (1989), 237.
5. Winicov, H., Schainbaum, J., Buckley, J., Longino, G., Hill, J. and Berkoff, C. E., *Chimica Acta,* **103** (1978), 469.
6. Legrand, M. and Bolla, P., *Journal of Automatic Chemistry,* **7** (1985), 31.
7. Chodosh, D. F., Wdzieckowski, F. E., Shainbaum, J. and Berkoff, C. E., *Journal of Automatic Chemistry,* **5** (1983), 99.
8. Frisbee, A. R., Nantz, M. H., Kramer, G. W. and Fuchs, P. L., *Journal of American Chemical Society.,* **106** (1984), 7143.
9. Ohfune, Y and Kurokawa, N., *Journal of Synthetic Organic Chemistry (Japan),* **44** (1986), 647.
10. Sangster, A. W., Thomas, S. E. and Tingling, N. L., *Tetrahedron,* **31** (1978), 878.
11. Kasi, T. and Sakamura, S., *Agricultural Biological Chemistry,* **45** (1981), 1483.
12. Miyazawa, T., *Bulletin of the Chemical Society* (Japan), **53** (1980), 2555 and 3661.

